# Evaluating Thresholds to Adopt Hypofractionated Preoperative Radiotherapy as Standard of Care in Sarcoma

**DOI:** 10.1155/2021/3735874

**Published:** 2021-10-26

**Authors:** Luca F. Valle, Nicholas Bernthal, Fritz C. Eilber, Jacob E. Shabason, Meena Bedi, Anusha Kalbasi

**Affiliations:** ^1^Department of Radiation Oncology, Jonsson Comprehensive Cancer Center and David Geffen School of Medicine, University of California, Los Angeles (UCLA), Los Angeles, CA, USA; ^2^Department of Orthopedic Surgery, Jonsson Comprehensive Cancer Center and David Geffen School of Medicine, University of California, Los Angeles (UCLA), Los Angeles, CA, USA; ^3^Departments of Surgery, Jonsson Comprehensive Cancer Center and David Geffen School of Medicine, University of California, Los Angeles (UCLA), Los Angeles, CA, USA; ^4^Department of Radiation Oncology, University of Pennsylvania, Philadelphia, PA, USA; ^5^Department of Radiation Oncology, Medical College of Wisconsin, Milwaukee, WI, USA

## Abstract

**Introduction:**

Data supporting hypofractionated preoperative radiation therapy (RT) for patients with extremity and trunk soft tissue sarcoma (STS) are currently limited to phase II single-institution studies. We sought to understand the type and thresholds of clinical evidence required for experts to adopt hypofractionated RT as a standard-of-care option for patients with STS.

**Methods:**

An electronic survey was distributed to multidisciplinary sarcoma experts. The survey queried whether data from a theoretical, multi-institutional, phase II study of 5-fraction preoperative RT could change practice. Using endpoints from RTOG 0630 as a reference, the survey also queried thresholds for acceptable local control, wound complication, and late toxicity for the study protocol to be accepted as a standard-of-care option. Responses were logged from 8/27/2020 to 9/8/2020 and summarized graphically.

**Results:**

The survey response rate was 55.3% (47/85). Local control is the most important clinical outcome for sarcoma specialists when evaluating whether an RT regimen should be considered standard of care. 17% (8/47) of providers require randomized phase III evidence to consider hypofractionated preoperative RT as a standard-of-care option, whereas 10.6% (5/47) of providers already view this as a standard-of-care option. Of providers willing to change practice based on phase II data, most (78%, 29/37) would accept local control rates equivalent to or less than those in RTOG 0630, as long as the rate was higher than 85%. However, 51.3% (19/37) would require wound complication rates superior to those reported in RTOG 0630, and 46% (17/37) of respondents would accept late toxicity rates inferior to RTOG 0630.

**Conclusion:**

Consensus building is needed among clinicians regarding the type and threshold of evidence needed to evaluate hypofractionated RT as a standard-of-care option. A collaborative consortium-based approach may be the most pragmatic means for developing consensus protocols and pooling data to gradually introduce hypofractionated preoperative RT into routine practice.

## 1. Introduction

Radiation therapy (RT) is an integral component of limb-salvage therapy for patients with high-risk soft tissue sarcoma (STS) [1]. RT can be delivered either preoperatively or postoperatively, though preoperative RT is generally favored [2] owing to advantages in long-term complications (at the expense of increased wound complication rates) [3]. The standard preoperative RT regimen is delivered in 25 fractionated treatment sessions over five weeks. However, given the logistical challenges [4–6] and psychological burden [7] of undergoing conventionally fractionated RT, especially for patients seeking care at sparsely distributed tertiary high-volume sarcoma centers, there has been growing interest in more condensed hypofractionated approaches to preoperative RT for STS.

Single-institution phase II data have emerged [8] suggesting that a five-fraction hypofractionated course of preoperative RT for STS may be safe alternative, with rates of major wound complications, fibrosis, joint stiffness, and lymphedema which are comparable to studies of conventionally fractionated RT [9–12]. While mature local control data are awaited, initial reports are promising. Several other hypofractionated approaches, including a 15-fraction regimen, are also being evaluated (Table 1) [13]. Regardless of the precise duration, shorter RT regimens have been shown to increase RT utilization and consolidate care at high-volume sarcoma centers [9].

Despite the appeal of a hypofractionated approach that is also potentially more cost effective [14], additional data are needed to support its inclusion in any major consensus guideline [15–17]. This presents a particular challenge for orphan diseases such as sarcoma, where large prospective multi-institutional phase 3 randomized studies are not always feasible [18]. Here, we set out to establish the type, threshold, and prioritization of evidence required by sarcoma experts before including hypofractionated preoperative RT as a guideline-based alternative for STS treatment. To do so, we designed a survey around a theoretical prospective single-arm multi-institutional study, which we used to evaluate specific target clinical endpoints among survey respondents.

## 2. Methods

Experts in the management of soft tissue sarcoma were identified either by word-of-mouth, through authorship of high-impact publications in sarcoma, through lead design of ongoing clinical trials in sarcoma, or by reviewing the websites of sarcoma centers of excellence.

We created a survey to assess characteristics of respondents, including oncologic specialty, practice location, and baseline views on whether 5-fraction preoperative RT is currently considered standard of care. Subsequently, the survey presented a theoretical, large (*N* > 250), multi-institutional, single-arm phase II study of 5-fraction preoperative RT, based on a recent single-institution phase II study [9]. No further details regarding this theoretical study were provided. The survey then assessed the respondents' thresholds for acceptable local control, wound complication, and late toxicity in this theoretical study, assuming the study protocol were to be accepted as a standard-of-care alternative. Late toxicities were defined as grade ≥2 fibrosis (RTOG/EORTC), joint stiffness (RTOG/EORTC), or edema (Stern's scale). For each clinical endpoint, the respondents were first prompted with data from RTOG 0630 [19] as a reference. Finally, respondents were asked to prioritize the clinical endpoints in order of importance for determining whether the study protocol would be accepted as a standard-of-care alternative.

The survey was created using Google Forms (Mountain View, CA) and transmitted electronically to sarcoma experts. The survey was open and accepted responses from August 27, 2020, at 12 : 00 pm Pacific Time (PT) until September 8, 2020, at 11 : 59 pm PT. Experts were sent a reminder e-mail prior to survey closure. An answer to each question was mandatory before advancing to the next survey question, though free-form text options were available for some questions. Free-form answers that were identical to preset answer choices were merged for final presentation of results. A copy of the full survey is available in the supplemental materials. This survey study was IRB approved (#20-001313, XXXX FWA00004642) prior to survey distribution.

## 3. Results

**Figure 1:**
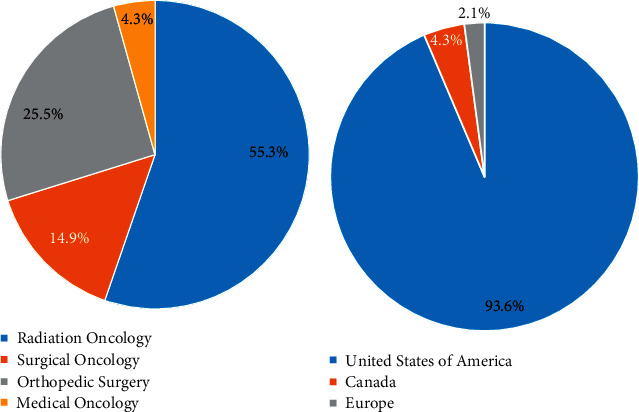
Demographics of surveyed providers. Breakdown of oncologic specialty (a) and practice location (b) among 47 survey respondents.

Out of 85 survey recipients, 47 sarcoma specialists completed the survey, for a response rate of 55.3%. The majority of respondents were radiation oncologists (55.3%, 26/47). Surgeons (surgical and orthopedic oncologists) comprised 40.4% of respondents, and medical oncologists comprised 4.3% (2/47) of respondents (Figure 1). The vast majority of respondents practice in the United States (93.6%, 44/47), compared to 4.3% (2/47) and 2.1% (1/47) in Europe and Canada, respectively.

**Figure 2:**
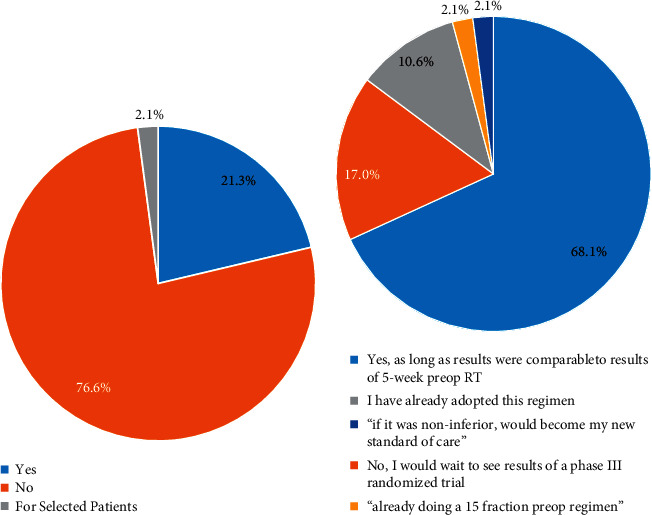
Viewpoints on the role of single-arm multisite phase II studies in changing radiotherapy standard of care in sarcoma. (a) Respondents (*n* = 47) answered whether they currently consider 5-fraction preoperative RT to be a standard-of-care option. (b) Respondents (*n* = 47) answered whether they would be willing to adopt a 5-fraction preoperative RT regimen if a sufficiently powered single-arm multisite phase II trial met their preferred clinical endpoint. Free-form responses are not included in the color-coded legend.

At baseline, 21.3% of respondents (10/47) already considered a 5-fraction preoperative RT regimen as a standard-of-care option (Figure 2(a)). One additional respondent (2.1%) considered this regimen as standard “for selected patients.” When asked if they would be willing to adopt a 5-fraction preoperative RT regimen if a theoretical multi-institutional phase II study was sufficiently powered (*N* > 250) and met their preferred clinical endpoint, 68.1% (32/47) stated they would accept phase II data, as long as results were comparable to results from a 5-week preoperative trial (Figure 2(b)). Fewer providers (17%, 8/47, including one free-form response) indicated that they would require randomized phase III data to change their practice. Notably, 10.6% (5/47) indicated that they have already adopted a 5-fraction preoperative regimen.

**Figure 3:**
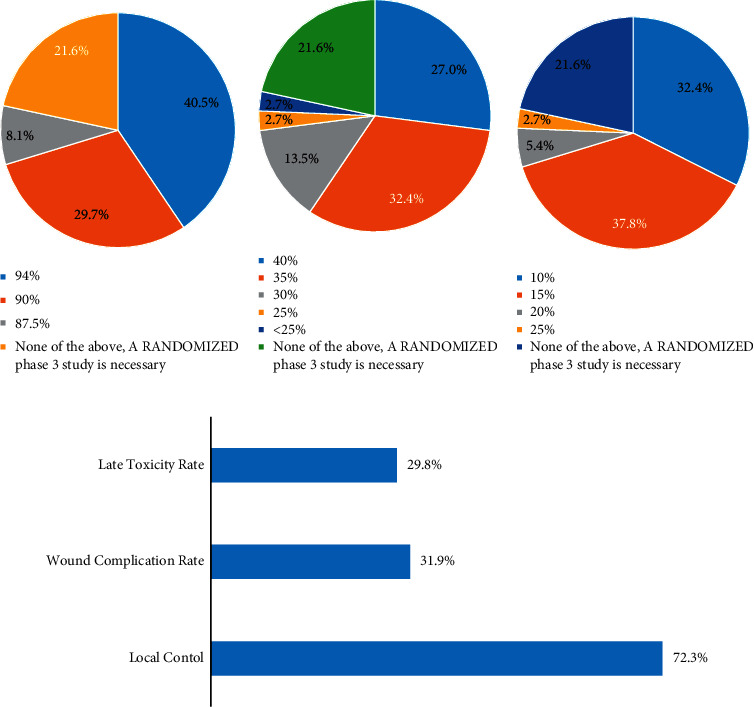
Optimal clinical trial endpoints and thresholds. Survey respondents (*n* = 37) were asked to provide their (a) local control, (b) wound complication, and (c) late toxicity thresholds for incorporating 5-day preoperative RT as a standard-of-care option. (d) Respondents (*n* = 47) prioritized the most important among these three endpoints. Free-form responses are not included in the color-coded legend.

Using the theoretically designed phase II study, we then evaluated thresholding of clinical endpoints (local control, wound complications, and late toxicity) among the 37 respondents who did not already consider preop hypofractionated RT to be a standard-of-care option. Respondents were first primed with local control results from the 73 patients who underwent standard 5-week preoperative RT and limb-salvage surgery on RTOG 0630 as a reference (94% [95% CI 88.2–99.7%]). A majority of providers (78.3%, 29/37) indicated they would either accept local control rates equivalent to those in RTOG 0630 (40.5%, 15/37, including one free-form comment) or local control rates less than those in RTOG 0630 but higher than 85% (37.8%, 14/37, including two free-form comments) (Figure 3(a)). The remaining physicians (21.6%, 8/37) indicated that they would require randomized phase III data to incorporate 5-fraction preoperative RT as a standard-of-care approach.

When primed with the wound complication rate from 71 evaluable patients on RTOG 0630 as a reference (36.6%), survey respondents had more conservative attitudes regarding acceptable wound complication rates. To incorporate 5-fraction preoperative RT as a standard-of-care approach, 51.3% (19/37, including two free-form responses) of respondents would require a wound complication rate superior to that reported in RTOG 0630; only 27% (10/37) would accept a slightly higher wound complication rate than reported in RTOG 0630 (Figure 3(b)). The remaining respondents (21.6%, 8/37) indicated that they would require randomized phase III data to incorporate 5-fraction preoperative RT as a standard-of-care approach.

Respondents were then primed with the late toxicity rate among 57 evaluable patients from RTOG 0630 (10.6%). To incorporate 5-fraction preoperative RT as a standard-of-care approach, 32.4% (12/37, including one free-form response) of respondents would require lower late toxicity rates compared to RTOG 0630 and 46% (17/37) of respondents would accept a late toxicity rate inferior to RTOG 0630 (Figure 3(c)). The remaining respondents (21.6%, 8/37) indicated that they would require randomized data to incorporate 5-fraction preoperative RT as a standard-of-care approach.

Among the three clinical endpoints assessed, local control was most frequently chosen by respondents as the endpoint most important in determining whether 5-fraction preoperative RT would be considered a standard-of-care option (Figure 3(d)). Additional comments provided by respondents at the end of the survey are provided in Supplementary Table 1.

## 4. Discussion

We report surveyed perspectives from sarcoma experts regarding hypothetical clinical trial endpoints that, if met, would be sufficient to enable the inclusion of hypofractionated preoperative RT as a standard-of-care option. We found that just 17% of providers require randomized phase III evidence to consider hypofractionated preoperative RT as a standard-of-care option. Moreover, respondents indicated that a large, single-arm multisite phase II study would be sufficient to incorporate this approach as a standard-of-care option. In fact, 10.5% of providers already use this approach despite the availability of only single-institution phase II nonrandomized studies. Finally, local control is the most important clinical outcome for sarcoma specialists when evaluating whether a radiotherapy regimen should be considered standard of care, though there is variability around the precise local control threshold.

Based on our data, there is no strong consensus on how to best evaluate hypofractionated preoperative RT as a standard-of-care option for the treatment of sarcoma. While our survey predominantly reflects opinions from experts in North America and is likely impacted by regional biases, it nonetheless highlights an important need for early consensus building through networks of sarcoma experts as a first step toward engineering new therapeutic approaches. Here, we discuss several potential pathways for evaluating this patient-centered and value-driven radiation treatment as a standard-of-care option.

The first pathway would be a multisite randomized phase III trial assessing the noninferiority of hypofractionated preoperative radiotherapy compared to conventionally fractionated preoperative radiotherapy. This approach has been the gold standard for changing practice in oncology. However, several hurdles, both logistical and financial [18], temper enthusiasm for this approach. For example, poor accrual might be a problem if patients are apprehensive about undergoing shorter courses of radiation. Furthermore, we anticipate a high rate of dropout in patients not randomized to the hypofractionated arm, especially in patients with limited means for traveling great distances to a tertiary sarcoma center [20]. While a study could be designed to include community radiation therapy centers, a potential lack of sarcoma care expertise could add variability to results. An alternative approach would be a multi-institutional, randomized, phase II trial, which would garner both credibility and generalizability and may be more feasible due to the smaller scale. However, many of the logistical challenges of randomization would apply in this scenario as well.

Another concern is that despite the power of a properly accrued randomized trial approach, such a design still may not offer a conclusive clinical answer, potentially due to the heterogeneous biologic profiles [21] of soft tissue sarcoma. For example, the recently published EORTC-62092: STRASS trial [22], a well-designed and executed randomized study, did not provide a conclusive answer to the clinical question of preoperative radiotherapy for retroperitoneal sarcoma [23–25].

Moreover, the high risk of a randomized study is unlikely to accompany high reward in this setting, given our survey results suggesting that over 10% of surveyed sarcoma experts already consider 5-fraction preoperative radiation to be a standard approach. Thus, as with other areas of research [26], the priorities involved in advancing treatments for rare diseases such as sarcoma should weigh both the costs and benefits of randomization.

A multi-institutional, nonrandomized, phase II trial is an alternative approach with a potential for faster accrual. Such a study would enhance the current evidence base surrounding hypofractionation, which mostly relies on single-institution studies. However, even if local control and wound toxicity were found to be on par with historical standards, the benefits of such a trial are again uncertain in the setting of widespread preexisting enthusiasm for preoperative hypofractionation off-study and the heterogeneous thresholds among clinicians.

A more grassroots approach might involve establishing a consortium of institutions that are comfortable performing hypofractionated RT in service of pooling outcomes from these centers so that we can continue to learn about the benefits and tradeoffs of this approach outside of the confines of a rigorous clinical trial protocol. This approach would be cost effective, promote knowledge and experience sharing among sarcoma experts, and would obviate the need for centralized IRB requirements. The obvious shortcoming here is that variable practices across centers may make data pooling and subsequent interpretation a challenge. This might be proactively managed by developing consensus protocols that aim to optimize the endpoints highlighted by this study as important for sarcoma experts, prior to inception of the consortium.

Finally, the most passive strategy would entail pausing research efforts and relying on natural forces and incentives [27] to drive a paradigm shift toward hypofractionation. This low-cost, low-effort approach has been replicated to some extent in other disease sites [28] and in tumors with similar biologic alpha/beta profiles [18, 29]. However, this strategy negates efforts to rigorously study patients treated with preoperative hypofractionation and leaves physicians ill-equipped to anticipate, counsel, and manage recurrences and toxicity appropriately.

We eagerly anticipate the long-term results of several completed and ongoing single-institution studies of hypofractionated RT. As discussed above, paradigm-shifting studies for radiotherapy can be challenging to orchestrate in sarcoma, and our survey results indicate the need for consensus building among clinicians on the type and threshold of evidence needed to evaluate hypofractionated RT as a standard-of-care option. One avenue will be cooperative-group sponsored multisite studies (e.g., single-arm phase 2 or randomized phase 2). However, given the existing obstacles around large, multisite, and randomized studies, the most pragmatic way forward may be a collaborative consortium-based approach for developing consensus clinical protocols with an eye toward pooling data from these individual institutional efforts.

## Figures and Tables

**Table 1:** Ongoing preoperative hypofractionated trials.

Study ID	Study title	Study design	Hypofractionated preoperative dose/fractionation	Status	Location	Institution
NCT04425967	Short course of preoperative radiotherapy in head and neck, trunk, and extremity soft-tissue sarcomas (SCOPES)	Randomized phase II study	14 Gy × 3	Recruiting	The Netherlands	Leiden University Medical Center
NCT02634710	Hypofractionated preoperative radiation therapy for soft-tissue sarcomas of the extremity and chest wall	Phase II study	7 Gy × 5	Active, not recruiting	United States	Medical College of Wisconsin
NCT03972930	Hypofractionated radiotherapy for soft-tissue sarcomas	Phase II study	Variable	Recruiting	United States	Medical College of Wisconsin
NCT04330456	Combined treatment of patients with soft-tissue sarcoma including preoperative stereotactic radiation therapy and postoperative conformal radiation therapy	Phase II study	5 Gy × 5	Recruiting	Russia	N.N. Petrov National Medical Research Center of Oncology
NCT02812654	Ifosfamide, doxorubicin, and hypofractionated radiotherapy in neoadjuvant sarcoma treatment	Phase II study	5 Gy × 5	Unknown	Brazil	A.C. Camargo Cancer Center
NCT03989596	Hypofractionated radiotherapy with hyperthermia in unresectable or marginally resectable soft-tissue sarcomas (SINDIR)	Phase II study	3.25 Gy × 10	Active, not recruiting	Poland	Maria Sklodowska-Curie Institute
NCT03819985	Shorter-course, hypofractionated presurgery radiation therapy in treating patients with localized, resectable soft-tissue sarcoma of the extremity of the superficial trunk	Phase II study	2.85 Gy × 15	Recruiting	United States	MD Anderson Cancer Center
NCT03651375	Hypofractionated radiotherapy with sequential chemotherapy in marginally resectable soft-tissue sarcomas of the extremities or trunk wall (UN-RESARC)	Phase II study	5 Gy × 5	Active, not recruiting	Poland	Maria Sklodowska-Curie Institute
NCT03816475	Hypofractionated radiotherapy in locally advanced myxoid liposarcomas of the extremities or trunk wall (LIPO-MYX trial) (LIPO-MYX)	Phase II study	5 Gy × 5	Active, not recruiting	Poland	Maria Sklodowska-Curie Institute
NCT02701153	Phase II study of 5-day hypofractionated preoperative radiation therapy for soft-tissue sarcomas: expansion cohort	Phase II study	5-6 Gy × 5	Recruiting	United States	UCLA
NCT04562480	Hypofractionated radiation therapy before surgery for the treatment of localized, resectable soft-tissue sarcoma of the extremity and superficial trunk	Phase II study	2.85 Gy × 15	Recruiting	United States	Mayo Clinic
NCT04617327	Preoperative RadiothErapy for soft-tissue SarcOmas (PRESTO)	Phase I/II study	7 Gy × 5	Recruiting	Canada	McGill University
NCT00822848	Sorafenib, epirubicin, ifosfamide, and radiation therapy followed by surgery in treating patients with high-risk stage II or stage III soft-tissue sarcoma	Phase I study	3.5 Gy × 8	Completed	United States	Oregon Health Sciences University
NCT03989596	Hypofractionated radiotherapy with hyperthermia in unresectable or marginally resectable soft-tissue sarcomas (SINDIR)	Phase II study	3.25 Gy × 10	Active, not recruiting	Poland	Maria Sklodowska-Curie Institute
NCT04398095	Radiotherapy with hyperthermia in recurrent and radiation-induced sarcomas (HOT)	Phase II study	3 Gy × 12	Recruiting	Poland	Maria Sklodowska-Curie Institute
NCT04506008	Hypofractionated radiotherapy followed by immediate surgical resection in the treatment of soft-tissue sarcomas	Phase II study	Not listed	Recruiting	United States	Vanderbilt University

## Data Availability

Survey data are available upon request.

## Supplementary Materials

Supplementary Materials Supplemental Table 1. Free-form comments from survey respondents on the need for a randomized study to evaluate hypofractionated preoperative radiation therapy as a potential standard of care. Supplemental Material 1: complete survey questionnaire.
