# Supplementary material for: Evaluating Thresholds to Adopt Hypofractionated Preoperative Radiotherapy as Standard of Care in Sarcoma
Source: Sarcoma. 2021 Oct 26;2021:3735874. doi: 10.1155/2021/3735874 (PMC8556117; doi:10.1155/2021/3735874)

**Supplemental Table 1:** Additional free-form Comments. Comments in green generally support a non-randomized study approach, comments in red generally support a randomized study approach, and comments in black are neutral on the subject of randomization.


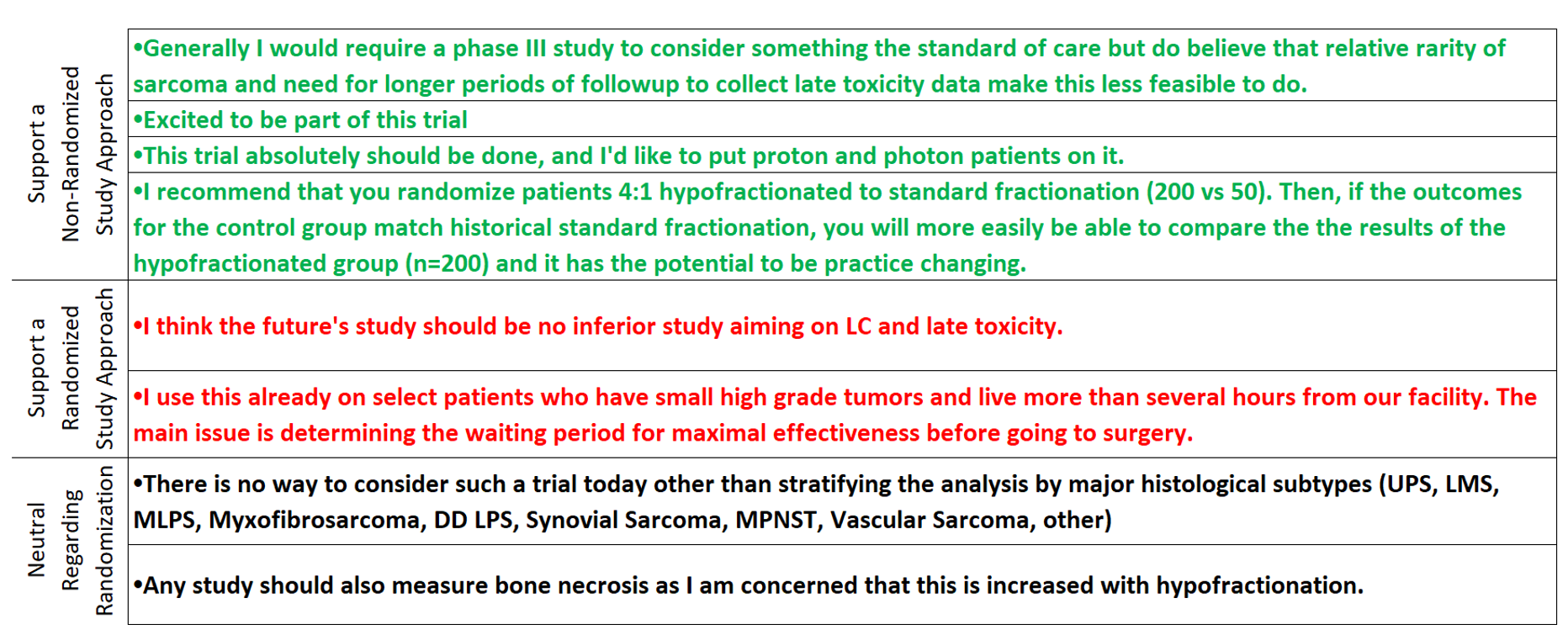


**Supplemental Material 1:** Complete Survey Questionnaire.


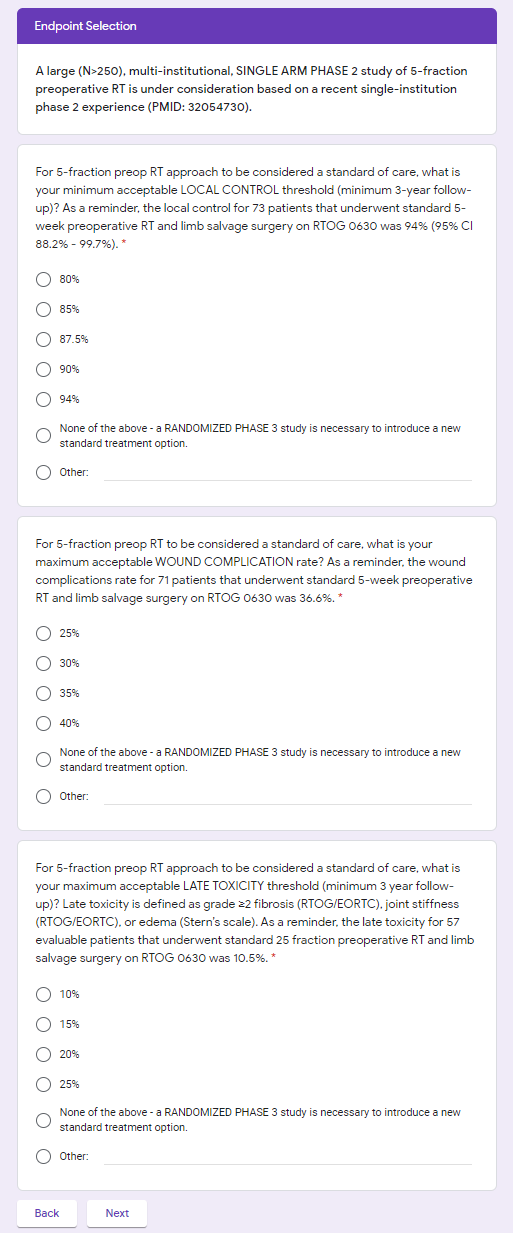

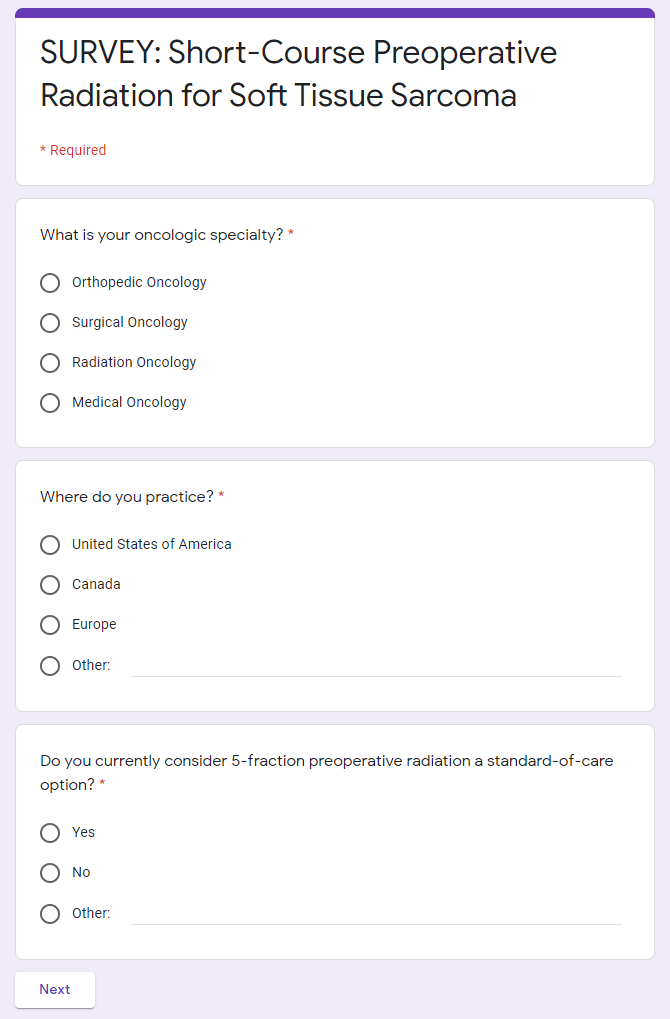


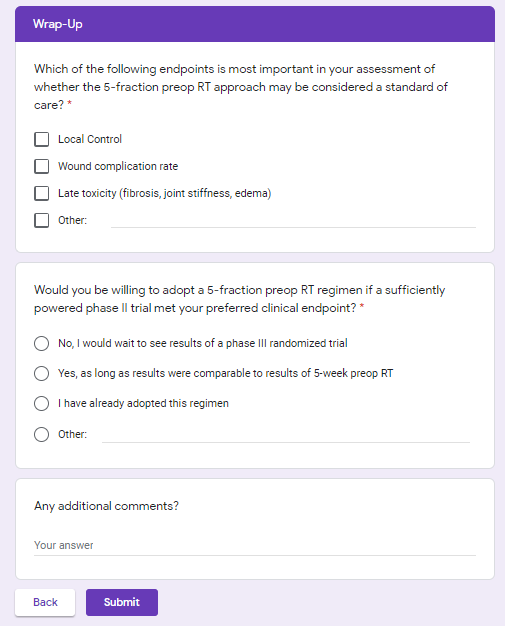

Supplement: Supplementary Materials — Supplemental Table 1. Free-form comments from survey respondents on the need for a randomized study to evaluate hypofractionated preoperative radiation therapy as a potential standard of care. Supplemental Material 1: complete survey questionnaire. [file 3735874.f1.docx]
